# Finger stick blood collection for gene expression profiling and storage of tempus blood RNA tubes

**DOI:** 10.12688/f1000research.8841.2

**Published:** 2017-03-03

**Authors:** Darawan Rinchai, Esperanza Anguiano, Phuong Nguyen, Damien Chaussabel

**Affiliations:** 1 Systems Biology Department, Sidra Medical and Research Center, Doha, Qatar; 2 The Jackson Laboratory for Genomic Medicine, Farmington, USA; 3 Baylor Scott and White Health, Dallas, Texas, USA

**Keywords:** Blood collection, Fingerstick, Gene expression, RNA, Tempus, Transcriptome

## Abstract

With this report we aim to make available a standard operating procedure (SOP) developed for RNA stabilization of small blood volumes collected via a finger stick. The anticipation that this procedure may be improved through peer-review and/or readers public comments is another element motivating the publication of this SOP. Procuring blood samples from human subjects can, among other uses, enable assessment of the immune status of an individual subject via the profiling of RNA abundance using technologies such as real time PCR, NanoString, microarrays or RNA-sequencing. It is often desirable to minimize blood volumes and employ methods that are the least invasive and can be practically implemented outside of clinical settings. Finger stick blood samples are increasingly used for measurement of levels of pharmacological drugs and biological analytes. It is a simple and convenient procedure amenable for instance to field use or self-collection at home using a blood sample collection kit. Such methodologies should also enable the procurement of blood samples at high frequency for health or disease monitoring applications.

## Introduction

Finger stick blood collection is a practical and minimally invasive sample collection method that is used for a wide range of applications in routine clinical practice and can be implemented outside of clinical settings. It is for instance by this means that millions of individuals collect daily small blood volumes to monitor sugar levels.

More recently, availability of high throughput profiling technologies made it possible to measure simultaneously the abundance of tens of thousands of analytes. For instance, transcriptome profiling, which measures abundance of RNA on a genome-wide scale has become a mainstay in biomedical research settings ^1– 6^. This approach can be implemented through the use of technologies such as microarray and more recently RNA-sequencing. Robust and more cost-effective “meso-scale” profiling technologies, relying for instance on PCR or NanoString probes, can profile the abundance of hundreds of genes ^7^. Blood transcriptome profiling has proven useful in generating high-resolution molecular phenotypes: to investigate pathogenesis of a wide range of diseases ^8– 11^; to develop biomarker signatures ^12– 15^; and to assess response to vaccines or therapies ^7, 16– 20^. More recently, an approach consisting in correlating serial blood transcriptome signatures with clinical course of disease was described as a means to guide development and selection of novel therapeutic modalities in patients with systemic lupus erythematosus ^1, 21^.

Transcriptome profiling studies have initially employed peripheral blood mononuclear cells (PBMCs) ^11, 22, 23^. PBMCs are isolated by fractionation and are enriched in blood leukocytes. It is also a type of sample from which high quality RNA can be reliably obtained, which at the time was not the case of whole blood. However, the PBMC preparation procedure involves multiple steps and important variations are introduced between the time of blood draw and preparation of the cell lysates ^24^. Furthermore, it is a time consuming process that requires trained personnel and equipment and is not straightforward to implement in most clinical settings. Whole blood RNA stabilization systems; PAXgene™ (Qiagen) and Tempus™ (Life Technologies) have been adopted as they became available and are now widely used. Several studies have compared the performance of these 2 commercial kits and found differences in gene expression profiles, RNA quality and yield ^25– 27^. Reported yields and quality of RNA stabilized in Tempus solution was generally greater. Thus, the choice of RNA stabilizing reagent used to preserve samples can indeed be important and affect subsequent RNA quantity and quality. Our choice of the tempus system over PaxGene dates from side-by-side comparisons we have performed over 10 years ago. However the vast majority of the studies carried out to date use relatively large volumes of venous blood ^12, 13, 19, 20, 28^. Collection of small volumes of blood via finger sticks is especially indicated for high frequency sample collection to enable monitoring of the immune status of individuals in health and disease ^2^. Advantages of this collection modality stem from the fact that it is less invasive, faster and does not require a trained phlebotomist. Therefore, it is more amenable to field applications and in home self-collection for proximity testing. A study by Obermoser *et al.,* employed this collection method to investigate transcriptome responses elicited by influenza and pneumococcal vaccines at 8 different time points in the 48 hours following vaccine administration ^16^. A methods development article has also been published by Robinson *et al.,* demonstrating that RNA quality and gene expression data obtained from blood obtained via finger stick (70 μL) and venipuncture (2.5 mL) are highly comparable ^29^. Since version 1 of our SOP came out in F1000Research ^30^ we have published a paper describing results of a study in which weekly in home self-finger stick blood collection was undertaken by 13 subjects with type 1 diabetes and 14 controls for a period of 6 months ^31^. Subjects returned an average of 24 out of 26 total weekly samples, and transcript data were successfully obtained for >99% of samples returned. A high degree of correlation between finger stick data and data from a standard 3 mL venipuncture sample was observed. RNA yields obtained from blood volumes ranging from 10, 15, 20, and 25 μL indicated that those volumes were sufficient to generate the 100 ng of RNA needed for high throughput real time-PCR ^31^. However, the detailed procedure for finger stick blood collection and RNA stabilization employed in this and other studies has never been published.

The standard operating procedure that we are sharing with this report will be published as part of our molecular profiling of pregnancy channel. Indeed, it was specifically developed for collection and stabilization of 50 μl of blood collected via a finger stick in a pregnancy monitoring study currently being conducted on the Thai-Myanmar border. It will consist of measuring changes in blood transcript abundance in 400 women during the second and third trimester of their pregnancy. A complete description of this study will be provided elsewhere.

### Narrative of the procedure

The procedure described in this article can be employed for serial blood collection in clinical or research laboratory settings as well as for in-home self-collection. A narrative is provided here, along with general remarks and considerations. A detailed point-by-point SOP follows.

Narrative: Tempus RNA tubes are designed for the collection of 3 ml of blood via venipuncture and contain 6 ml of a proprietary RNA stabilizing reagent. For the collection of 50 μl blood samples 100 μl of the RNA stabilizing reagent is aliquoted in microfuge tubes. Blood is collected with a plastic capillary straw. Immediately after collection, the tube is shaken vigorously to disrupt the blood cells for at least 20 seconds. A sufficient quantity of RNA for downstream processing was obtained when tubes were flicked, pipetted, or vortexed, but was reduced when samples were not mixed at all. Lysis of blood cells occurs upon thoroughly mixing the blood drawn into the tube and the stabilizing reagent. Furthermore, RNases are inactivated and the freed RNA is selectively precipitated and thus further protected from degradation. Effective stabilization of the RNA ensures that the transcriptional profile is maintained and will accurately reflect the physiological state of the patient at the time of the blood draw. RNA properly collected in Tempus solution and stored at -20°C or -80°C will remain stable for minimum of 6 years ^32^.

General remarks: After over 10 years of use across a wide range of clinical settings RNA stabilization using tempus solution has in our hands proven robust and reliable. However there are a few things that we have learned that are worth sharing:

1) Finger stick: The finger is usually the preferred site for capillary testing in an adult patient. When samples are collected serially it is recommended to choose a different finger from the one used for the last procedure to prevent bruising. The sides of the heel are only used in pediatric and neonatal patients. The guidance given in Section 7.1 of the WHO guidelines on blood drawing: best practices in phlebotomy, can help decide whether to use a finger or heel-stick, and with the selection of an appropriately sized lancet ^33^.

2) Blood volumes: The volume can be adjusted depending on the application. Typical yield from 50 μl of blood is the minimum about 500 ng of total RNA. Procedures for RNA extraction and quality control will be shared in a separate publication (Anguiano E., Rinchai D., Tomei S., Chaussabel D., unpublished report). A study was conducted where as little as 15 μl of blood was collected, which was sufficient to run a high throughput Fluidigm PCR assay ^31^. Such small blood volumes can also be obtained serially from mice, which allow longitudinal monitoring of individual animals. In human studies, instead of using a capillary straw small blood volumes can also be collected and measured with a micropipette. The blood is then placed into the microfuge tube containing the tempus solution. This can be done when collecting small volumes of blood from a finger stick or obtaining a small aliquot of blood from a larger venous blood draw.

3) Volume of RNA stabilization solution: The appropriate ratio of [Blood : RNA stabilizing reagent] is 1 volume of blood for 2 volumes of tempus solution (in our case 50 μl of blood in 100 μl of RNA stabilizing reagent). Loss in RNA quality and quantity will be observed if this ratio is not respected. Collecting more blood will actually result in decreased yields and RNA quality. In cases when the amount of blood collected is lower, the volume of tempus solution can be adjusted accordingly when feasible. The same ratio can be used when working with mouse blood collected from the tail vein using a similar procedure (as mentioned above blood volumes can be lowered to 15 μl). The volumetric ratio is usually lower when working with non-human primate species (e.g. 1:3, 1:4) and should be determined on a species-by-species basis (a 1:3 ratio is used when collecting blood from macaques ^34^).

4) Sample mixing: This, after maintaining an appropriate blood: tempus solution ratio, is the second most critical aspect of the procedure, and a potential cause of sample failure. As mentioned above samples must be homogenized by thorough mixing in order to disrupt cells and release their RNA cargo. The RNA will precipitate in the tempus solution and in this form is protected from degradation by the RNAses that are present in the sample.

5) Temperature: RNA should remain in a precipitated state at “room temperature”. Although refrigeration and freezing at the earliest possible time is recommended, based on our observations keeping the blood lysates at room temperature (25°C) for up to 24 hours should not affect RNA quality. Samples can be stored at 4°C (refrigerator or cold packs) for up to 48 hours, which can simplify the logistics associated with temporary storage, transfer and shipping of samples post-collection. Based on information provided by the manufacturer RNA should remain in a precipitated state as long as temperatures remain below 30°C. It may therefore be necessary to take precaution when working in warm climates.

6) Storage and shipping: After collecting the blood sample should be kept cold at 4°C no longer than 48 hours and transferred to a -20°C or -80°C freezer for long-term storage. By default, samples are stored in the lab at -20°C. Data obtained using a limited set of samples frozen overnight showed that the RNA yield for samples stored at -80°C was about half the yield of same blood samples stored at -20°C, but was nevertheless still amply sufficient for downstream analyses. It should also be noted that the plastic tempus tubes are made of will become brittle at temperatures lower than -20°C. The effect of storage temperature on RNA yield and quality will have to be evaluated further, especially over extended periods of time where storage at lower temperatures might show benefits (see also referees comments and our response for more details). Shipments are made on dry ice although for overnight shipping in cooler climates using ice packs should be sufficient (however testing using mock samples is recommended). When shipping “off the shelf” tempus tubes direct contact with dry ice should be avoided to prevent breakage. When shipping on dry ice attention should be paid to the thickness of the walls of the polystyrene container holding the tubes along with the dry ice. The thinner the walls the faster the shipment will run out of dry ice. This is especially important to consider when contemplating longer transit times and/or warm weather conditions. Regarding biosafety, we have found the tempus solution to prevent growth of bacteria known for their resilience such as *Burkholderia pseudomallei* (Rinchai D, unpublished report), and thus conclude that threat of contamination via tempus blood lysates is likely to be low. However, appropriate testing should be carried out on a case-by-case basis and all procedures in the laboratory involving tempus lysates should be consistent with standard blood handling procedures.

7) Safety: The hazardous nature of the tempus solution would make extensive roll out of the collection procedure described in this manuscript problematic and is at the moment clearly intended for research use under well controlled conditions, with preferably collection carried out by trained personnel. However, it should be noted that it has been-field tested for in-home self-collection in a limited number of subject over a period of 6 months without incident ^31^. The fact that small volumes of solution are used may alleviate some concerns (30 microliters of solution for 15 microliters of blood in the above-mentioned study, vs 6 ml of solution for 3 ml of blood using “off the shelf” tempus collection tubes). However, other technical solutions in which liquids are better contained may indeed be preferable (e.g. microfluidics card, sponges), with one of the best example being the recently developed “DxCollect” system (DxTerity, Rancho Dominguez, CA).

## Materials and methods

### Reagents and equipment

- Tempus ^TM^ blood RNA tubes (ThermoFischer Scientific, Waltham, MA, USA; Product number 4342792; https://www.thermofisher.com/order/catalog/product/4342792)
- Capillary blood and tube assembly, untreated, 50 μl, thin design (Kabe Labortechnik, Nümbrecht, Germany (Product number GK100, http://www.kabe-labortechnik.de/download/kapillarblut_en.pdf) ^35, 36^
- Sterile blood lancets
- Alcohol pads
- Biohazard container
- Lab coat
- Gloves
- Ziploc-type biohazard bag and freezer box
- Adhesive bandages
- Sample collection tube labels

## Preparation of sample collection tubes

**Figure 1.**
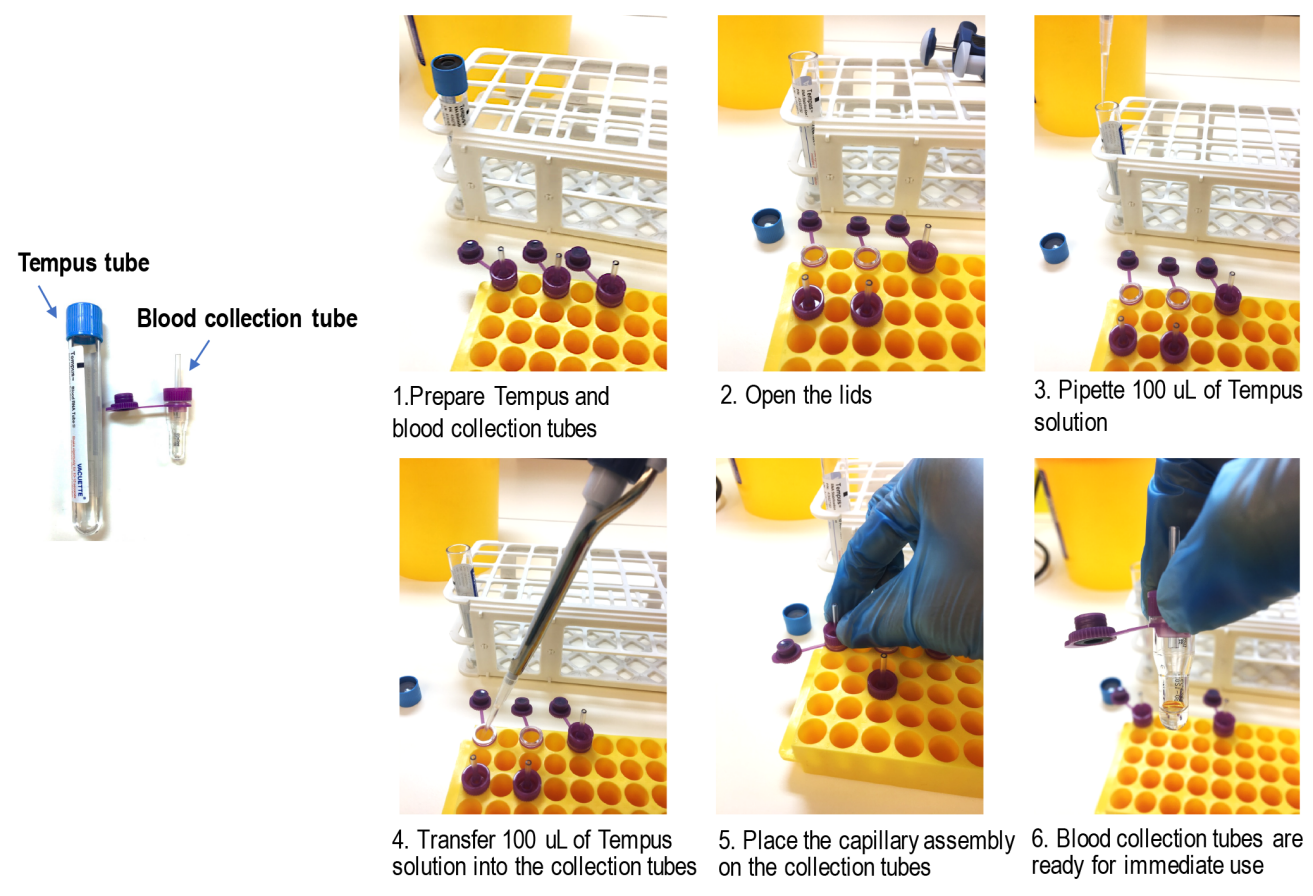
Preparation of the sample collection tubes. This figure illustrates the different steps involved in preparation sample collection tubes for collection and stabilization of blood RNA.

1. Prepare Tempus Tube RNA stabilization into blood collection tubes ( Figure 1)
  a. Place the blood collection tubes on a rack and remove the capillary assembly and place them at the same rack.
  b. Put the Tempus tube on a second rack and remove the lid
  c. Pipette 100 ul of RNA stabilizing solution from the open tempus tube and transfer it into the blood collection tubes
  d. Place the capillary assembly on the collection tube. The blood collection tubes are ready for immediate use. In case the blood collection tubes are prepared for later use the lid should be closed and capillary assembly set aside.
  e. Store the blood collection tubes containing RNA stabilizing solution in storage box at room temperature
2. For the later use, assembly capillary tube and blood collection tubes
  a. Open the lid of blood collection tubes containing the RNA stabilizing solution
  b. Assemble the capillary assembly into the collection tubes containing the RNA stabilising solution
3. Follow the blood sample collection procedure step-by-step

## Precautions

- Personal protective equipment (PPE) must be worn to prevent accidental exposure to blood and bloodborne pathogens, and to help reduce contamination during sample collection [ http://www.cdc.gov/niosh/topics/emres/ppe.html].
- Discard all blood collection materials and “sharps” in properly labeled biohazard containers approved for their disposal.
- Check that the liquid preservatives and anticoagulants in the collection tubes are clear and colorless. Do not use any tubes if they are discolored or contain precipitates.
- Tempus Tube RNA stabilization reagent is a potential health hazard; acute oral toxicity, skin corrosion/irritation and serious eye damage/eye irritation can occur upon contact (See MSDS for details).

## Procedures

**Figure 2.**
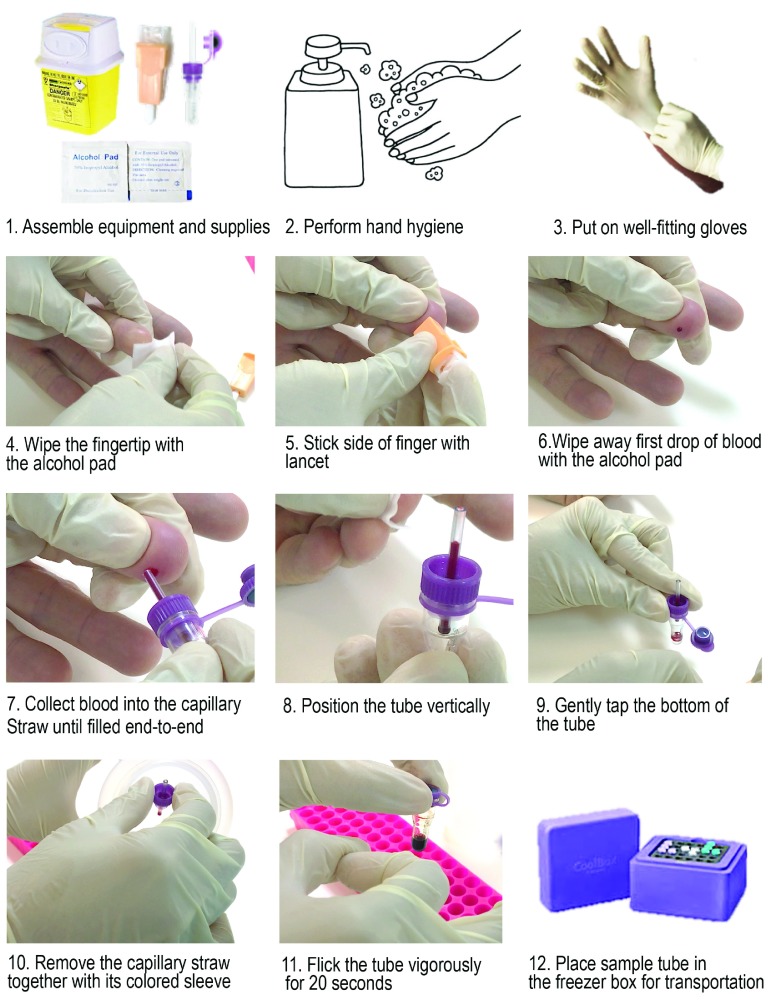
Illustration for capillary blood sampling. This figure illustrates the different steps involved in capillary blood sampling via finger stick.

The procedures below are illustrated in Figure 2 and a demonstration video is available here: https://www.youtube.com/watch?v=Mxc36ARxWqQ

1. Assemble equipment and supplies, then complete the finger stick information log by recording relevant information about blood collection such as patient name, patient identity number (patient ID), date of blood collection, frequency number of blood collection (Day 1 ^st^, Day 7 ^th^... Day 90 ^th^,…). Double check that the label on the collection tube matches with the patient ID.
2. Put on well-fitting gloves
3. Choose one of the subject’s fingers from which blood will be collected. The middle or the ring finger is the best choice for finger stick collection, ideally of the non-dominant hand. Fingers on the non-dominant hand are generally less calloused. Avoid the thumb that may be calloused and has pulse and pinkie finger that is often calloused and potentially more sensitive to pain due to additional nerve ending, fingers with thick calluses, that are injured or swollen and fingers with tight rings as they may constrict blood flow.
4. Prepare the puncture site by warming the area. If the subject is particularly cold have the subject wash hands under warm water to stimulate blood flow. In addition, it may be necessary to warm the area with a moist towel for five to ten minutes.
5. Wipe the fingertip with the alcohol pad and allow to air-dry completely without blowing or wiping off the alcohol.
6. To stimulate blood flow, you may shake or gently knead the subject’s hand from palm to fingertip. Blood will also flow better if the hand is kept lower, approximately at the level of the subject’s waist.
7. Hold finger and press lancet firmly against the side of the center of the finger, with lancet oriented perpendicular to the fingerprint grooves.
8. Discard lancet in an appropriate container.
9. Release pressure and allow a full drop of blood to collect on finger. If necessary, gently knead the palm only to stimulate blood flow.
10. Wipe away the first drop of blood with a sterile gauze pad because it may be contaminated with tissue fluid or debris (sloughing skin).
11. Collect blood sample into the capillary tube.
  a. Hold the capillary and micro-tube assembly horizontally, and touch the tip of the capillary to the blood drop.
  b. The blood will be pulled into the tube via capillary action.
  c. Be sure to allow the capillary to fill end-to-end to allow collection of accurate blood volume.
  d. To expel the sample from the capillary, place the capillary and micro-tube assembly vertically and firmly tap the bottom of the tube. Remove capillary tube together with cap assembly system and discard in the appropriate biohazard container.
  e. **It is important to maintain the appropriate blood sample to tempus solution ratio.** A volume of 50 μl of blood should be added to the 100 μl of tempus solution. If necessary the volume of solution can be adjusted to the available or desired volume of blood; e.g. for 15 μl of blood use 30 μl of tempus solution.
  f. Close the micro-tubes, making sure that the cap is pressed down firmly to avoid any spillage during sample homogenization.
  g. To prevent clotting, blood samples should be collected within 30 seconds of performing the finger stick. Clotted samples will not be usable.
12. Have the subject apply pressure to the puncture site using sterile gauze pad until bleeding has stopped and apply a bandage. Do not use the alcohol pad as contact of an open wound with alcohol would be painful for the subject.
13. Immediately, mix the blood sample and preservative thoroughly by holding the top of the tube between thumb and index of one hand and **flicking the tube vigorously for 20 seconds with the index finger of the other hand ( Figure 2)**. This is important because it will allow precipitation of the RNA, which is then protected from degradation.
14. If not already in place stick pre-printed label with sample information on the sample tube.
15. Place sample tube in appropriate container (e.g. freezer box).
16. The sample should be kept cold at 4°C no longer than 48 hours and transferred to a -20°C or -80°C freezer for long-term storage. Note that RNA integrity is still preserved when samples are kept at “room temperature” for a few hours as long as the temperature does not rise above 30°C.
17. For local transportation samples can be kept in a freezer box containing ice or ice packs. For international shipping samples can be kept on dry ice in a freezer box.
