# Supplementary material for: Finger stick blood collection for gene expression profiling and storage of tempus blood RNA tubes
Source: F1000Res. 2017 Mar 3;5:1385. Originally published 2016 Jun 15. [Version 2] doi: 10.12688/f1000research.8841.2 (PMC5357033; doi:10.12688/f1000research.8841.2)
Supplement: Peer review history

# Peer review: Referee response for version 2

**DOI:** 10.5256/f1000research.11887.r20676

**Published:** 2017-03-10

**Authors:** Eiliv Lund

**Affiliations:** 1 Department of Community Medicine, The Arctic University of Norway, Tromsø, Norway

Dear Authors, you have a remarkable work to improve the manuscript which I find clear and with better instructions. You have addressed my concerns adequately. The future could bring us better technologies still.

I have read this submission. I believe that I have an appropriate level of expertise to confirm that it is of an acceptable scientific standard.

# Peer review: Referee response for version 1

**DOI:** 10.5256/f1000research.9516.r16392

**Published:** 2016-10-14

**Authors:** Eiliv Lund

**Affiliations:** 1 Department of Community Medicine, The Arctic University of Norway, Tromsø, Norway

This manuscript describes an important approach to transcriptomics that could be important for future research. It is remarkable that modern technology can give mRNA profiles based on microgram of mRNA. The SOP is important, but some parts of it confuse me.

 First of all, this company is related to Tempus. Can the method be extended to other products? This is important since PAX is as much used as Tempus.

 Secondly, I am a bit surprised that -20 degrees is the primary choice versus -70 degrees since all medical biobanks use -70 degrees. The explanation of plastic that is brittle does not sound convincing for future use of the technology.

 Third, how long is the optimal mixing time before freezing? You state 20 seconds in the illustration while you also write that it can be stored for 24 hours at normal temperatures.

 The technology is very interesting. Since there are no comparisons of outcomes for different procedures nor any quality measures of mRNA it may look more like a recipe.

I have read this submission. I believe that I have an appropriate level of expertise to confirm that it is of an acceptable scientific standard, however I have significant reservations, as outlined above.

# Response

**Published:** 2017-02-23

**Authors:** Darawan Rinchai

**Affiliations:** Sidra Medical and Research Center, Qatar

*We thank the reviewer for their precious spent reviewing our manuscript. Please see our point by point response below.*

 This manuscript describes an important approach to transcriptomics that could be important for future research. It is remarkable that modern technology can give mRNA profiles based on microgram of mRNA. The SOP is important, but some parts of it confuse me.

**First **of all, this company is related to Tempus. Can the method be extended to other products? This is important since PAX is as much used as Tempus.

***Authors:*** *Our choice of the tempus system over PaxGene is based on side-by-side comparisons we have performed over 10 years ago.* *We have checked in publications whether PAXgene were used for small volume blood collection. We have found only a publication by Carrol ED. et al., BMC Immunol 2007 where the PAXgene Blood RNA System kit protocol was modified for used with small, sick children. These investigators aliquoted 860 uL of PAXgene reagent into microtubes and then added 300 uL whole blood to maintain the same recommended proportions as in the PAXgene evacuated tube system. Total RNA yield was between 1,114 and 2,950 ng. This blood volume is not compatible with finger stick blood collection but it is possible that a protocols very similar to the one we are publishing here could be derived for use with PAXgene solution instead of Tempus.*

*However, there are many studies have compared the “ **Differences in RNA quality and yield between PAXgene™ and Tempus™”.*** *Here are some selected publications; *

1. *Asare AL. et al., BMC Genomics 2008* *have reported that “* *the tempus system has higher mean yields, improved RNA purity based on OD 260/230 ratios, less degradation based on GAPDH 3'/5 ratios, and a higher number of expressed transcripts based on Percent Present Calls [PMID:* *18847473* *]. *
2. *Nikula T. et al., Transl Res 2013 have compared the performance of Tempus and PAXgene systems by using 2 RNA amplification protocols and high-density microarrays. “The m* *icroarray analysis showed acceptable correlation within and between the RNA preservation methods, but altogether 443 transcripts were differentially expressed between RNA samples preserved in TEMPUS and PAXgene tubes”. However, the TEMPUS gene expression profile more closely resembles the PBMC than the PAXgene [PMID:* *23138105]* *. *
3. *Häntzsch M. et al., PLoS One 2014. That have compared the 3 PAXgene and 3 Tempus tubes samples which collected from participants of the LIFE study with and without acute myocardial infarction (AMI). They extracted RNA with 4 manual protocols from Qiagen (PAXgene Blood miRNA Kit), Life Technologies (MagMAX for Stabilized Blood Tubes RNA Isolation Kit), and Norgen Biotek (Norgen Preserved Blood RNA Purification Kit I and Kit II), and 2 (semi-)automated protocols on the QIAsymphony (Qiagen) and MagMAX Express-96 Magnetic Particle Processor (Life Technologies). The results showed that RNA yields were highest using the Norgen Kit I with Tempus tubes and lowest using the Norgen Kit II with PAXgene [PMID:25469788].*

 
*Thus, choice of RNA stabilizing reagent used to preserve samples can indeed be important and affect subsequent RNA quantity and quality [(* *1) -(3)* *]* *. We have added sentences below to our introduction;*

*“Whole blood RNA stabilization systems; PAXgene™ (Qiagen) and Tempus™ (Life Technologies) have been adopted as they became available and are now widely used. Several studies have compared the performance of these 2 commercial kits and found differences in gene expression profiles, RNA quality and yield [Asare AL. et al., BMC Genomics 2008., Nikula T. et al., Transl Res 2013., Häntzsch M. et al., PLoS One 2014]. Reported yields and quality of RNA stabilized in Tempus solution was generally greater. Thus, the choice of RNA stabilizing reagent used to preserve samples can indeed be important and affect subsequent RNA quantity and quality. Our choice of the tempus system over PaxGene dates from side-by-side comparisons we have performed over 10 years ago.”*

**Secondly, **I am a bit surprised that -20 degrees is the primary choice versus -70 degrees since all medical biobanks use -70 degrees. The explanation of plastic that is brittle does not sound convincing for future use of the technology.

***Authors: This point was also addressed in response to Dr. Cliff’s comment:*** *Regarding to the optimal temperature for long term RNA storage. The preliminary data that we are referring to comparing the yield of RNA from samples that were storage at **-**20* *°* *C or -80* *°* *C are shown below **.***

**Table 1:** Preliminary results for comparison of -20 °C and -80 °C storage conditions

**Sample ID** **Storage Temp (°C)** **RNA yield (ug)** **RNA RIN**

       S1                        -20                                    8.59                          8.2

       S2                        -20                                    8.69                          8.0

       S3                        -80                                    3.04                          9.0

       S4                        -80                                    2.81                          8.9

       NC                                                                0.05                          N/A

**Note:** Two whole blood samples collected in Tempus RNA Tubes were combined into 50 mL conical tubes and then mixed thoroughly by inversion. Two aliquots of 4 mL each were stored at -80ºC or -20ºC for 3 days. Samples were subsequently processed using modified MagMAX for Stabilized Blood Tubes RNA Extraction Kit (adjusted for input volume during homogenization step only).  Purified RNA was assessed for concentration and integrity using NanoDrop and BioAnalyzer, respectively.  Negative control is a 1X PBS processed through RNA extraction workflow.

*The result shows that* *samples stored at -20* *°* *C have greater RNA yield compared to those stored at -80* *°* *C, but that quality was similar under both storage conditions. It is conceivable that over the long term integrity however would be better maintained at the lower temperature. We feel sharing these preliminary results at this point could be helpful to some readers although more extensive investigation is required, especially with long-term storage – although yields and QC results will obviously not be available for some time.*

*However, when using off the shelf vacuutainer tempus tubes* *it has indeed been our experience that tubes in direct contact with dry ice can break during transportation and will shatter if dropped to the ground. Recommendation for -20* *°* *C storage were made by the manufacturer on this basis. However, this is not a concern when using microtubes in which small volumes of blood in tempus solution are stored. The appropriate sentences were added into the manuscript (6) storage and shipping).*

**Third,** how long is the optimal mixing time before freezing? You state 20 seconds in the illustration while you also write that it can be stored for 24 hours at normal temperatures.

***Authors:*** *The 20 seconds mixing step is necessary and should always be performed. It should occur immediately after blood collection. This is important because it will allow precipitation of the RNA, which is then protected from degradation. We have clarified this point in the manuscript.*

*Furthermore, in a recent paper by Speake C, et al.,* *Clin Exp Immunol 2016* *in which we report the implementation of a procedure using 15 ul of fingerstick for in home blood collections we demonstrated that quality of RNA was similar when tubes were flicked, pipetted, or vortexed, but was reduced when samples were not mixed at all.*

*Subsequently to this homogenization step we recommend that samples be stored: *

- *at room temperature for a few hours; *
- *at 4°C for no longer than 48 hours; *
- *at -20°C or -80°C freezer for long-term storage.*

*It should be noted that per the manufacturer samples could be stored for 5 days at room temperature but in our experience this can lead to significant loss in RNA integrity and it is generally best to refrigerate or freeze as soon as feasible.*

 The technology is very interesting. Since there are no comparisons of outcomes for different procedures nor any quality measures of mRNA it may look more like a recipe.

***Authors:*** *We indeed tried to be pragmatic and publish a detailed SOP with sufficient level of details that it could be directly incorporated in a clinical protocol. Outcome comparisons with RNA quality and quantity measurements are presented in a paper that was published very recently and is now referred to in version 2 of our manuscript.*

# Peer review: Referee response for version 1

**DOI:** 10.5256/f1000research.9516.r16014

**Published:** 2016-10-04

**Authors:** Jacqueline Margaret Cliff

**Affiliations:** 1 Department of Immunology and Infection, London School of Hygiene and Tropical Medicine, London, UK

Thank you for the opportunity to review the manuscript by Rinchai *et al*, entitled “Finger stick blood collection for gene expression profiling and storage of tempus blood RNA tubes”.

 Blood gene expression profiling has led to rapid advances in our understanding of a range of pathological conditions, including cancer, autoimmune disease and infectious disease. Blood RNA stabilisation tubes have greatly advanced the ease and standardisation of blood collection for such studies. Developing this methodology further by enabling reduced blood volume collection by non-trained phlebotomists or even study participants themselves would facilitate more detailed investigations at greater frequency and more pertinent time points, for example during times of disease exacerbation, and by removing the necessity to visit healthcare settings.

 This article describes such a procedure, describing the collection of 50µl blood in a capillary tube, collected into Tempus Tube stabilisation reagent. This paper therefore provides an important contribution to the blood transcriptomics field. However, the manuscript could be improved if the following points are addressed.

1. The authors are suggesting that this sample collection method could be rolled out for home-based testing. However, the Tempus Tube RNA stabilisation reagent is hazardous according to the MSDS. This should be mentioned in the manuscript under the precautions section, whilst also addressing this limitation in terms of suitability for extensive roll-out for personal sample collection.
2. Whilst the manuscript, including the figure and the accompanying video, are very explicit about the blood sample collection into the capillary tube, the preparation of the sample collection tubes in advance is not described. Including this under the preparation steps would be very helpful, as from the video it is difficult to see the Tempus reagent already in the tube.
3. The paper would be substantially improved by the inclusion of some RNA quality data, although the authors indicate RNA extraction will be published in a separate manuscript. But it would be reassuring to see some quantity and quality indicators in this manuscript, or the inclusion of some downstream analysis data to demonstrate that reasonable quality RNA can be generated.

I agree with reviewer Angela Vinturache that the paper does not show much data regarding the storage of Tempus tubes as described in the paper title, except for a note about storage at -20 ^o^C being better than -80 ^o^C. In our experience, we get good yields of RNA from Tempus Tubes when samples have been stored at -80 ^o^C, as normally recommended for long-term RNA storage, believed to be due to inactivation of RNAses.  A comment on why -20 ^o^C is better would be useful.

 Overall I think this is an important manuscript which could stimulate the expansion of blood-based transcriptomics for disease analysis and treatment monitoring, and recommend that it is indexed, subject to addressing the reservations (particularly point 1) described above.

I have read this submission. I believe that I have an appropriate level of expertise to confirm that it is of an acceptable scientific standard, however I have significant reservations, as outlined above.

# Response

**Published:** 2017-02-23

**Authors:** Darawan Rinchai

**Affiliations:** Sidra Medical and Research Center, Qatar

*We thank the reviewer for their precious spent reviewing our manuscript. Please see our point by point response below.*

 Blood gene expression profiling has led to rapid advances in our understanding of a range of pathological conditions, including cancer, autoimmune disease and infectious disease. Blood RNA stabilisation tubes have greatly advanced the ease and standardisation of blood collection for such studies. Developing this methodology further by enabling reduced blood volume collection by non-trained phlebotomists or even study participants themselves would facilitate more detailed investigations at greater frequency and more pertinent time points, for example during times of disease exacerbation, and by removing the necessity to visit healthcare settings.

 This article describes such a procedure, describing the collection of 50µl blood in a capillary tube, collected into Tempus Tube stabilisation reagent. This paper therefore provides an important contribution to the blood transcriptomics field. However, the manuscript could be improved if the following points are addressed.

 1. The authors are suggesting that this sample collection method could be rolled out for home-based testing. However, the Tempus Tube RNA stabilisation reagent is hazardous according to the MSDS. This should be mentioned in the manuscript under the precautions section, whilst also addressing this limitation in terms of suitability for extensive roll-out for personal sample collection. 

***Authors:*** *We thank reviewer for this suggestion. RNA stabilizing reagent is indeed potentially hazardous and designed for research use only. We therefore added the paragraph below under the “precaution” and “general remarks” sections, respectively and as suggested discuss this point as well.*

***“*** *Tempus Tube RNA stabilization reagent is a potential health hazard; acute oral toxicity, skin corrosion/irritation and serious eye damage/eye irritation can occur upon contact (See* *MSDS* *for details)”*

*The hazardous nature of the tempus solution would make extensive roll out of the collection procedure described in this manuscript problematic and is at the moment clearly intended for research use under well controlled conditions, with preferably collection carried out by trained personnel. However, it should be noted that it has been-field tested for in-home self-collection in a limited number of subject over a period of 6 months without incident [Speake C., et al., Clin Exp Immunol 2016]. The fact that small volumes of solution are used may alleviate some concerns (30 microliters of solution for 15 microliters of blood in the above-mentioned study, vs 6 ml of solution for 3 ml of blood using “off the shelf” tempus collection tubes). However, other technical solutions in which liquids are better contained may indeed be preferable (e.g. microfluidics card, sponges), with one of the best example being the recently developed “DxCollect” system (DxTerity, Rancho Dominguez, CA).*

 2. Whilst the manuscript, including the figure and the accompanying video, are very explicit about the blood sample collection into the capillary tube, the preparation of the sample collection tubes in advance is not described. Including this under the preparation steps would be very helpful, as from the video it is difficult to see the Tempus reagent already in the tube.

***Authors:*** *As suggested by the reviewer, we added a new section and figure (new Figure 1) for “Preparation of collection tubes” in “Materials and Methods” section. We provide the step-by-step procedure for aliquoting Tempus RNA stabilization and preparation of the blood collection tubes.*

  3. The paper would be substantially improved by the inclusion of some RNA quality data, although the authors indicate RNA extraction will be published in a separate manuscript. But it would be reassuring to see some quantity and quality indicators in this manuscript, or the inclusion of some downstream analysis data to demonstrate that reasonable quality RNA can be generated.

***Authors:*** *A proof of principle has been obtained with a* *study that we recently published, in which weekly in home self-finger stick blood collection (15 uL) was implemented for 13 subjects with type 1 diabetes and 14 controls for a period of 6 months [Speake C., et al., Clin Exp Immunol 2016]. A high degree of correlation between results obtained via fingerstick and a standard 3 mL venipuncture sample was observed. RNA yields obtained from blood volumes ranging from 10, 15, 20, and 25 μL indicated that those volumes were sufficient to generate the 100 ng of RNA needed for downstream high throughput real time-PCR. Furthermore, it was found that equivalent quantities of of RNA were obtained whether tubes were flicked, pipetted, or vortexed, but was reduced when samples were not mixed at all.*

*Several sentences were added into part of introduction and the work referenced throughout, where appropriate.*

*However, the initial experiment with RNA extraction of 50 uL blood collection protocol in Tempus solution, we obtained the average RNA yield was 1 ug, Purity of RNA; average of RNA integrity number (RIN) was ~7.* *5 and* *A260/A280 ratio of ~2.14. This concentration was enough for downstream assays; RNA sequencing, microarray or Fluidigm assay. These data will be included in our manuscript describing the RNA extraction procedures.*

 I agree with reviewer Angela Vinturache that the paper does not show much data regarding the storage of Tempus tubes as described in the paper title, except for a note about storage at -20 ^o^C being better than -80 ^o^C. In our experience, we get good yields of RNA from Tempus Tubes when samples have been stored at -80 ^o^C, as normally recommended for long-term RNA storage, believed to be due to inactivation of RNAses.  A comment on why -20 ^o^C is better would be useful.

***Authors:*** *We thank the reviewer for helping us to make our manuscript clearer. The following sentence has been added to the manuscript (6) Storage and shipping)*

*“After collecting the blood sample should be kept cold at 4°C no longer than 48 hours and transferred to a -20°C or -80°C freezer for long-term storage. Data obtained using a limited set of samples frozen overnight showed that the RNA yield for samples stored at -80°C was about half the yield of same blood samples stored at -20°C, but was nevertheless still amply sufficient for downstream analyses. It should also be noted that the plastic tempus tubes are made of will become brittle at temperatures lower than -20°C. The effect of storage temperature on RNA yield and quality will have to be evaluated further, especially over extended periods of time where storage at lower temperatures might show benefits (see also referees comments and our response for more details).”*

*We intend to publish standard operating procedures for RNA extraction from tempus blood, which will include extensive QA/QC analyses under different storage conditions. The preliminary data that we are referring to when comparing the yield of RNA from samples that were storage at **-**20* *°* *C or -80* *°* *C are shown below **.***

**Table 1:** Preliminary results for comparison of -20 °C and -80 °C storage conditions

**Sample ID** **Storage Temp (°C)** **RNA yield (ug)** **RNA RIN**

       S1                        -20                                    8.59                          8.2

       S2                        -20                                    8.69                          8.0

       S3                        -80                                    3.04                          9.0

       S4                        -80                                    2.81                          8.9

       NC                                                                0.05                          N/A

**Note:** Two whole blood samples collected in Tempus RNA Tubes were combined into 50 mL conical tubes and then mixed thoroughly by inversion. Two aliquots of 4 mL each were stored at -80ºC or -20ºC for 3 days. Samples were subsequently processed using modified MagMAX for Stabilized Blood Tubes RNA Extraction Kit (adjusted for input volume during homogenization step only).  Purified RNA was assessed for concentration and integrity using NanoDrop and BioAnalyzer, respectively.  Negative control is a 1X PBS processed through RNA extraction workflow.

*The result shows that* *samples stored at -20* *°* *C have greater RNA yield compared to those stored at -80* *°* *C, but that quality was similar under both storage conditions. We feel sharing these preliminary results as part of our point by point response could be helpful to some readers although more extensive investigation is required, especially with long-term storage – although yields and QC results will obviously not be available for some time.*

# Peer review: Referee response for version 1

**DOI:** 10.5256/f1000research.9516.r15424

**Published:** 2016-09-29

**Authors:** Angela E Vinturache

**Affiliations:** 1 Department of Physiology and Pharmacology, Alberta Children's Hospital Research Institute for Child and Maternal Health, Cumming School of Medicine, University of Calgary, Calgary, AB, Canada

Thank you for the opportunity to review the manuscript Finger stick blood collection for gene expression profiling and storage of tempus blood RNA tubes by Rinchai D, Anguiano E, Nguyen P and Chaussabel D.

 In this paper, the authors are proposing an SOP for finger stick blood collection for RNA profiling. 

 While the objective of this manuscript is worthwhile for the scientific world in proposing standardized lab methods, the paper would benefit from some improvement.  

 There is a disconnect between the title and the objective of the paper. Is the focus on the finger stick collection or on the tempus tube storage? This should be revised, especially considering that the authors intend to publish the RNA extraction in a separate paper.

**Introduction:**

 First sentence is unclear. The introduction could be shortened considerably. A more concise and to the point argument on why we need this method, advantages and disadvantages can be made. Also, the authors should add supporting reasons for why their method should be published and how it is different from the others.

 The authors mention that the method will be used in the future. Is this to understand they have no experience with the proposed method?

 Why is mentioning where/what study this method will be used important? Do authors have any experience with non-pregnant patients? Does pregnancy bear any weight in sample collection and transportation?

**Narrative of the procedure:**

 The figure is very useful for following the procedure step-by-step. Considering that a follow up paper proposed by the authors will discuss RNA extraction, all the practical points shared under 'General Remarks' could be moved to the respective manuscript. Since this paper proposes to discuss only the finger stick procedure, the discussion about the volumes of blood drawn is exhaustive and unwarranted for this particular manuscript. However, it is not clear if the tubes for the blood collection are the same as the tubes pre-prepared with the volume of RNA stabiliser for 50 μL of blood. Please provide some practical explanation on the procedure when the capillary straw is not filled (i.e. how to measure the blood volume). What type of capillary is to be used?

**Precautions:**

 There is no mention of the possibility of sample contamination. For point 3 and 5, I suggest they add the reasoning for the selected choices in addition to the drawbacks of the other possibilities. Eliminate repetitions about blood flow and the ratio blood to solution. Point 16 is quite vague. As the matter is essential for the RNA quality, I invite the authors to expand the topic and share their experience, with numbers rather than “as soon as possible”. How much of those samples could be kept during transportation? Some information on authors’ experience with the bio-repository would be very welcome.

 The authors mention somewhere about using the method as in home self collection. Have they experimented that? It would be particularly interesting if they would provide details about how they plan quality assurance for these samples. Also, any particularities of this method of collection should be explained.

I have read this submission. I believe that I have an appropriate level of expertise to confirm that it is of an acceptable scientific standard, however I have significant reservations, as outlined above.

# Response

**Published:** 2017-02-23

**Authors:** Darawan Rinchai

**Affiliations:** Sidra Medical and Research Center, Qatar

*We thank the reviewer for their precious spent reviewing our manuscript. Please see our point by point response below.*

 In this paper, the authors are proposing an SOP for finger stick blood collection for RNA profiling. While the objective of this manuscript is worthwhile for the scientific world in proposing standardized lab methods, the paper would benefit from some improvement. There is a disconnect between the title and the objective of the paper. Is the focus on the finger stick collection or on the tempus tube storage? This should be revised, especially considering that the authors intend to publish the RNA extraction in a separate paper.

**Introduction:**

 First sentence is unclear. The introduction could be shortened considerably. A more concise and to the point argument on why we need this method, advantages and disadvantages can be made. Also, the authors should add supporting reasons for why their method should be published and how it is different from the others.

 The authors mention that the method will be used in the future. Is this to understand they have no experience with the proposed method?

 Why is mentioning where/what study this method will be used important? Do authors have any experience with non-pregnant patients? Does pregnancy bear any weight in sample collection and transportation?

***Authors:*** *The introduction provides the rationale for adopting such a sample collection procedure. The benefits of this approach were mentioned in the third paragraph of the “Introduction”. We could not find a major disadvantage of this method, except quality and quantity of RNA may be affected if the protocol was to be adapted to be used collecting blood lower than 15 uL.*

*As per the reviewer’s suggestion,*

  1) *We have edited the first paragraph to make it more clear and concise.*

*2) A detailed standard operating procedure has not been provided elsewhere before, which is the reason why we are publishing this report (and to gather feedback from other users/reviewers). We have first used this approach 6 years ago, with ensuing publications (Obermoser* *., et al. 2013* *), and more recently in a study in which samples were collected from diabetics and control subjects in home and on a weekly basis* *(* *Speake C., et al., Clin Exp Immunol 2016* *)* *– however those details have not been published before* *. The intent was to publish a ready to use SOP that could be readily incorporated  in a clinical protocol. We have also added a sentence to clarify these points. * 

*3) Pregnancy has no particular bearing on this procedure, only this is mentioned here as the paper is published as part of our* *“* *molecular* *profiling of pregnancy channel”. We have added more information into this sentence.*

*"The standard operating procedure that we are sharing with this report will be published as part   of our molecular profiling of pregnancy channel. Indeed, it was specifically developed for      collection and stabilization of 50 μl of blood collected via a finger stick in a pregnancy monitoring study currently being conducted on the Thai-Myanmar border.* *”*

**Narrative of the procedure:**

 The figure is very useful for following the procedure step-by-step. Considering that a follow up paper proposed by the authors will discuss RNA extraction, all the practical points shared under 'General Remarks' could be moved to the respective manuscript. Since this paper proposes to discuss only the finger stick procedure, the discussion about the volumes of blood drawn is exhaustive and unwarranted for this particular manuscript. However, it is not clear if the tubes for the blood collection are the same as the tubes pre-prepared with the volume of RNA stabiliser for 50 μL of blood. Please provide some practical explanation on the procedure when the capillary straw is not filled (i.e. how to measure the blood volume). What type of capillary is to be used?

***Authors:*** *Discussion of volume is extensive and warranted as this is in our experience one of the main causes of failure during the extraction step; we now provide a procedure detailing “preparation of the sample collection tubes” (Please see “Material and Methods”); the capillary needs to be filled in order to collect appropriate volumes. Alternative methods, such as use of micropipettes is possible if preferred; other products are also available and could be tested for that purpose. We describe the method that in our hands produces the best results for our particular conditions.*

**Precautions:**

 There is no mention of the possibility of sample contamination.

***Authors:*** *Tempus ^TM^ Blood RNA stabilizing Reagent immediately lyse whole blood and stabilize RNA in single step. We have tested lysate in the solution by bacterial culture and found that the sample were still sterilized after blood collection. Therefore we never found contamination after blood collection. The only possibility of the contamination is from sterile/hygiene of blood collection. Personal protective equipment (PPE) is a key of sterile sample collection. We therefore added more details into point 1 of “Precaution” about the point of sample contamination as below.*

- *“Personal protective equipment (PPE) must be worn to prevent accidental exposure to blood and bloodborne pathogens, and to help of reducing contamination during sample collection [* *http://www.cdc.gov/niosh/topics/emres/ppe.html* *].”*

For point 3 and 5, I suggest they add the reasoning for the selected choices in addition to the drawbacks of the other possibilities. Eliminate repetitions about blood flow and the ratio blood to solution.

***Authors:*** *Ideally choosing a finger for fingerstick blood collection is a finger of non-dominant hand which are generally less calloused. We also need to choose the fingers that less painful for subjects. Therefore, the best choice should be middle and ring finger. We don’t recommend the thumb that may be calloused and has pulse and pinkie finger or index finger that is often calloused and potentially more sensitive to pain due to additional nerve ending.*

*We added this information in the manuscript.*

 Point 16 is quite vague. As the matter is essential for the RNA quality, I invite the authors to expand the topic and share their experience, with numbers rather than “as soon as possible”. How much of those samples could be kept during transportation? Some information on authors’ experience with the bio-repository would be very welcome.

***Authors:*** *As the comment by reviewer **, **we have edited point #16 “The sample should be kept cold at 4°C no longer than 48 hours and transferred to a -20°C or -80°C freezer for long-term storage.”*

 The authors mention somewhere about using the method as in home self collection. Have they experimented that? It would be particularly interesting if they would provide details about how they plan quality assurance for these samples. Also, any particularities of this method of collection should be explained.

***Authors:*** *Since version 1 of our SOP came out in F1000Research (F1000Research 2016, 5:1385 (doi: 10.12688/f1000research.8841.1) we have published a paper describing results of a study in which weekly in home self-finger stick blood collection was undertaken by 13 subjects with type 1 diabetes and 14 controls for a period of 6 months [Speake C., et al., Clin Exp Immunol 2016]. Subjects returned an average of 24 out of 26 total weekly samples, and transcript data were successfully obtained for >99% of samples returned. A high degree of correlation between finger stick data and data from a standard 3 mL venipuncture sample was observed.* *RNA yields obtained from blood volumes ranging from 10, 15, 20, and 25 μL indicated that those volumes were sufficient to generate the 100 ng of RNA needed for downstream high throughput real time-PCR.* *[Speake C., et al., Clin Exp Immunol 2016].  However, the detailed procedure for finger stick blood collection and RNA stabilization employed in this and other studies has never been published.*

 The paragraph above was included in the manuscript (Part of **Introduction**).

*This point was also addressed in response to Dr. Cliff’s comment:* *In* *Our initial experiment with RNA extraction of 50 uL blood collection protocol in Tempus solution, we obtained the average RNA yield was 1 ug, Purity of RNA; average of RNA integrity number (RIN) was ~7.* *5 and* *A260/A280 ratio of ~2.14. Such yields and quality are sufficient for downstream assays; including RNA sequencing, microarray or Fluidigm. These data will be included in our manuscript describing RNA extraction procedures.*
